# Time course profiling of host cell response to herpesvirus infection using nanopore and synthetic long-read transcriptome sequencing

**DOI:** 10.1038/s41598-021-93142-7

**Published:** 2021-07-09

**Authors:** Zoltán Maróti, Dóra Tombácz, Norbert Moldován, Gábor Torma, Victoria A. Jefferson, Zsolt Csabai, Gábor Gulyás, Ákos Dörmő, Miklós Boldogkői, Tibor Kalmár, Florencia Meyer, Zsolt Boldogkői

**Affiliations:** 1 Department of Pediatrics, Faculty of Medicine, University of Szeged, Somogyi B. u. 4., Szeged, 6720 Hungary; 2 Department of Medical Biology, Faculty of Medicine, University of Szeged, Somogyi B. u. 4., Szeged, 6720 Hungary; 3 MTA-SZTE Momentum GeMiNI Research Group, University of Szeged, Somogyi B. u. 4., Szeged, 6720 Hungary; 4 Department of Biochemistry and Molecular Biology, Entomology and Plant Pathology, Mississippi State University, 408 Dorman Hall, 32 Creelman St., Box 9655, Starkville, MS 39762 USA

**Keywords:** Gene expression analysis, Transcriptomics, Virus-host interactions, Transcriptomics, RNA sequencing, Gene expression profiling

## Abstract

Third-generation sequencing is able to read full-length transcripts and thus to efficiently identify RNA molecules and transcript isoforms, including transcript length and splice isoforms. In this study, we report the time-course profiling of the effect of bovine alphaherpesvirus type 1 on the gene expression of bovine epithelial cells using direct cDNA sequencing carried out on MinION device of Oxford Nanopore Technologies. These investigations revealed a substantial up- and down-regulatory effect of the virus on several gene networks of the host cells, including those that are associated with antiviral response, as well as with viral transcription and translation. Additionally, we report a large number of novel bovine transcript isoforms identified by nanopore and synthetic long-read sequencing. This study demonstrates that viral infection causes differential expression of host transcript isoforms. We could not detect an increased rate of transcriptional readthroughs as described in another alphaherpesvirus. According to our knowledge, this is the first report on the use of LoopSeq for the analysis of eukaryotic transcriptomes. This is also the first report on the application of nanopore sequencing for the kinetic characterization of cellular transcriptomes. This study also demonstrates the utility of nanopore sequencing for the characterization of dynamic transcriptomes in any organisms.

## Introduction

Bovine alphaherpesvirus type 1 (BoHV-1) is a large DNA virus belonging to the *Alphaherpesvirinae* subfamily. This virus infects cattle and causes the disease commonly known as bovine respiratory disease, which leads to severe economic losses annually worldwide^1^. Like other alphaherpesviruses, such as herpes simplex virus type 1 (HSV-1), or pseudorabies virus (PRV), BoHV-1 also enters a latent state most commonly in the trigeminal ganglia following primary infection^2^. From this state, the virus can be reactivated by various types of stress and can re-establish an acute infection^3^.

Short-read sequencing (SRS) technology has expanded the frontiers of genomic and transcriptomic research due to its capacity to collect vast quantities of sequencing data at a relatively low cost. However, the past decade has witnessed incredible advances in long-read sequencing (LRS) technology. Besides the Pacific Biosciences and Oxford Nanopore Technologies platforms, Loop Genomics has recently also developed an LRS technique based on single molecule synthetic long-read sequencing (LoopSeq). LRS approaches present a strategy that is able to elude the limitations of SRS, including its ineffectiveness in the identification of transcript isoforms and in distinguishing overlapping RNA molecules. Recently, LRS techniques have been widely applied for the transcriptome analysis of a variety of organisms^4–8^, including herpesviruses^9–12^. These studies have uncovered a far more complex transcriptional landscape of the examined species than previously thought. Genome-wide sequencing assays have annotated the global transcriptome of BoHV-1^13^, including microRNAs^14^. The effect of herpesvirus infection on host cell transcription using SRS (Illumina HiSeq) has been characterized by^15^. In this paper, the authors described alternative splicing and polyadenylation in human skin fibroblast cells due to the infection by HSV-1.

In this work, we carried out a time-lapse assay for the examination of the effect of BoHV-1 infection on host [bovine (*Bos taurus*)] cell gene expression. The transcriptome analysis was performed using MinION sequencing from Oxford Nanopore Technologies (ONT) and Illumina-based LoopSeq from Loop Genomics.

## Results

### Annotation of *Bos taurus* transcripts

In this work, we applied the following techniques for the analysis of bovine transcriptome: (1) direct cDNA sequencing (dcDNA-Seq) based on oligo(dT)-primed reverse transcription (RT), (2) amplified cDNA sequencing based on random-oligonucleotide-primed RT using nanopore sequencing on ONT MinION platform, as well as (3) synthetic long-read sequencing (LoopSeq) on Illumina platform. All of the three techniques were used for bovine transcript annotation, whereas dcDNA-Seq was used for the time-varying analysis of the effect of BoHV-1 on host cell gene expression. For transcript detection and annotation, mapped reads were analyzed using the LoRTIA software suite developed in our laboratory (https://github.com/zsolt-balazs/LoRTIA).

For the annotation of introns, transcription start sites (TSSs), and transcription end sites (TESs), we set the criterion that these sequences have to be identified by the LoRTIA suit in at least two independent bovine cell samples. With this restriction, we identified altogether 11,025 TSSs, 21,317 TESs and 139,771 introns (Supplementary Table S1). Additionally, LoRTIA produced a total of 227,672 bovine transcripts (Supplementary Data Item 1). The median length of these transcripts was 1678 nt (σ = 2386.5).

Three biological replicates were prepared for each time-point in dcDNA sequencing used for the time-lapse experiment. Seven time points post infection (p.i.) and a mock-infected sample was used in each replicate for this part of the analysis (Supplementary Fig. S1).

**Figure 1:**
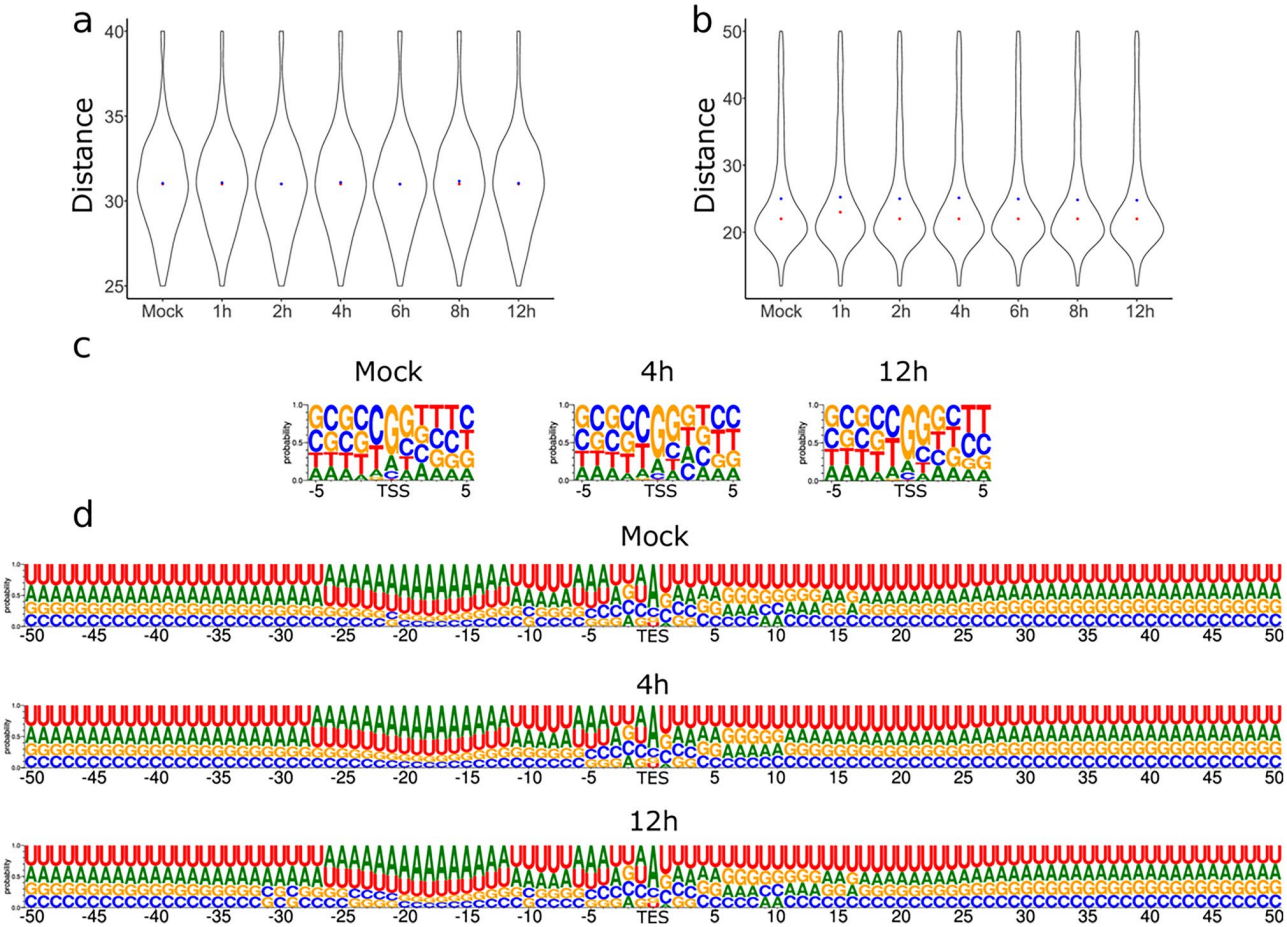
The effect of viral infection on transcript start and end sites. (**a**) The distance of TATA boxes from TSSs and (**b**) the distance of polyadenylation signals from TESs in base pairs. The sequence motif of the (**c**) TSSs and the (**d**) TESs in the mock infected cells, 4 h and 12 h following infection. Panels (a) and (b) were created using R v. 3.6.3 (https://www.r-project.org/) and ggplot2 v. 3.3.3 (https://ggplot2.tidyverse.org/reference/ggplot.html). Panels (**c**) and (**d**) were created using the online tool WebLogo3 (http://weblogo.threeplusone.com/). The figure was created using Inkscape v. 0.92 (https://inkscape.org/).

We identified consensus TATA boxes at a mean distance of 31.15 nt (σ = 2.96) upstream of bovine TSSs. The polyadenylation signals (PASs) were located at a mean distance of 25.35 nt (σ = 8.26) upstream of the host TES. Our data show that viral infection does not induce significant changes in the distance between promoters and TSSs as well as between PASs and TESs (Fig. 1a, b). No significant modification was found in the sequence of the ± 5 nt surrounding region of the TSS and the ± 50 nt surrounding region of bovine gene TESs during the infection (Fig. 1c, d).

**Figure 2:**
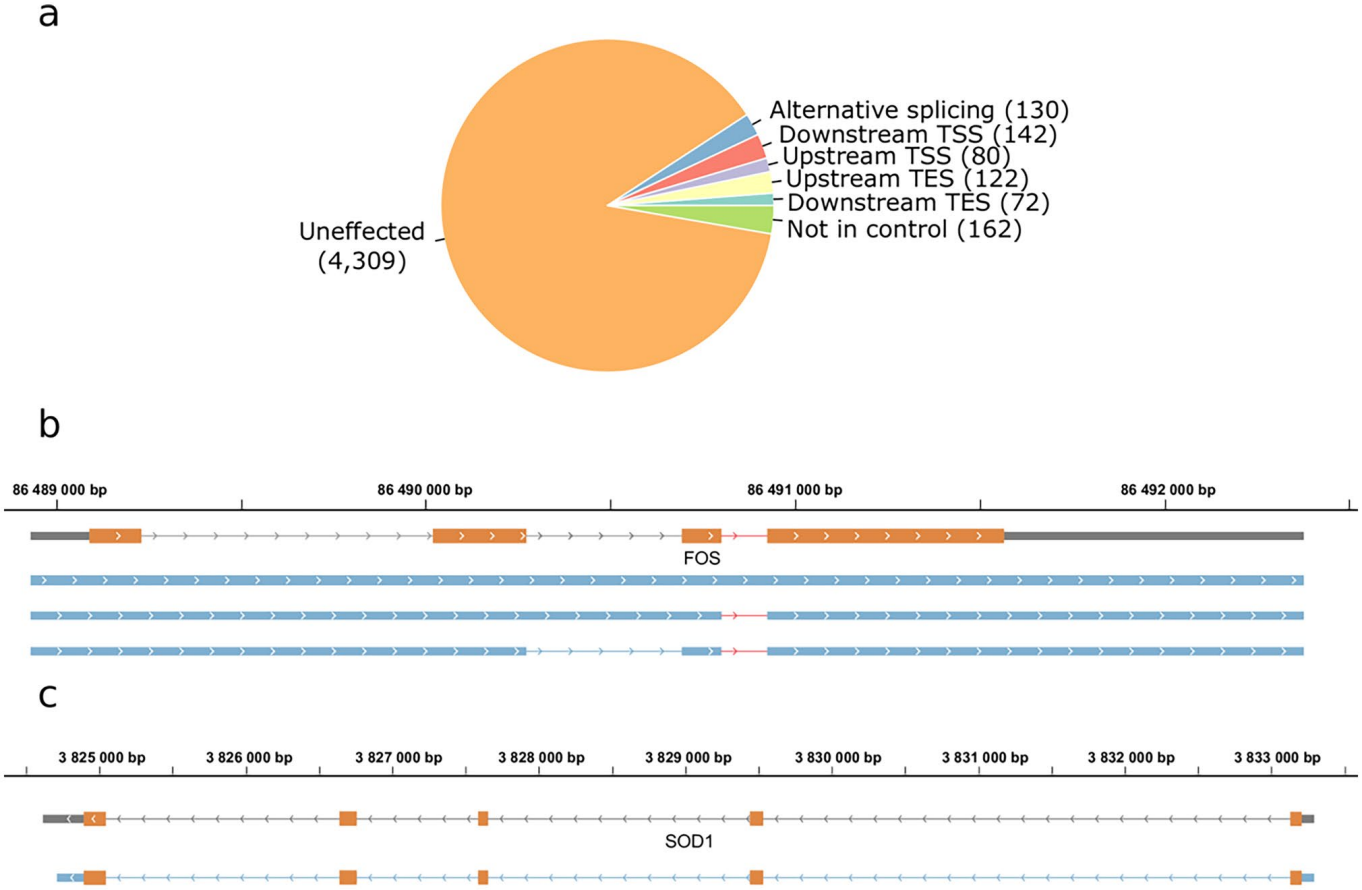
The effect of viral infection on the transcript isoforms of host cells. (**a**) A total of 546 transcript isoforms were detected with at least ten reads in the datasets of transcripts derived from infected bovine cells compared to the transcripts present in the mock dataset. (**b**) Gray rectangles represent previously detected transcript isoforms. The blue rectangles represent a novel non-spliced and two new splice variants of the FOS transcript. Introns are represented by lines between rectangles. The intron which is responsible for the increased degradation rate of FOS is shown in red. Orange rectangles represent the ORF. (**c**) The blue rectangles represent the novel TES isoform of SOD1, the lines represent introns, and orange rectangles illustrate the ORF. A SOD1 isoform with an upstream TES detected during the infection but not in the mock samples. Panel (**a**) was created using R v. 3.6.3 (https://www.r-project.org/). Panel (**b**) and (**c**) was created using IGV (https://software.broadinstitute.org/software/igv/). The figure was created using Inkscape v. 0.92 (https://inkscape.org/).

To assess changes in splicing, and the usage of TSSs and TESs of the host cell during BoHV-1 infection, we evaluated transcripts represented by more than ten reads in the infected samples (n = 69,726) reported by LoRTIA. We detected altogether 130 alternatively spliced transcripts (Fig. 2a).

FOS, an immediate responder of the stress signaling pathway, is quickly degraded if its third intron is retained^16^. We detected a non-spliced variant of FOS in very low abundance and additional splice variants of the transcript lacking the above-mentioned exon, which were present starting from the first hour of the infection (Fig. 2b). This confirms previous reports on the presence of FOS in the early stages of viral infections^15,17^.

The 3′-UTRs of genes often contain miRNA targets, contributing to mRNA degradation. Thus, shorter 3′-UTR length can lead to increased transcript stability^18^, whereas longer 3′-UTRs can be targeted by several miRNAs and other trans-acting elements thereby generating distinct regulation patterns^19^. We detected 72 transcripts with TESs located further downstream and 122 transcripts with TESs located more upstream compared to transcripts in mock samples. Superoxide dismutase 1 (SOD1) confers protection against oxidative damage^20^, including that induced by the IFN-I signaling^21^. A 3′-UTR isoform of SOD1 detected in infected cells was shorter than that of found in the mock sample (Fig. 2c).

**Figure 3:**
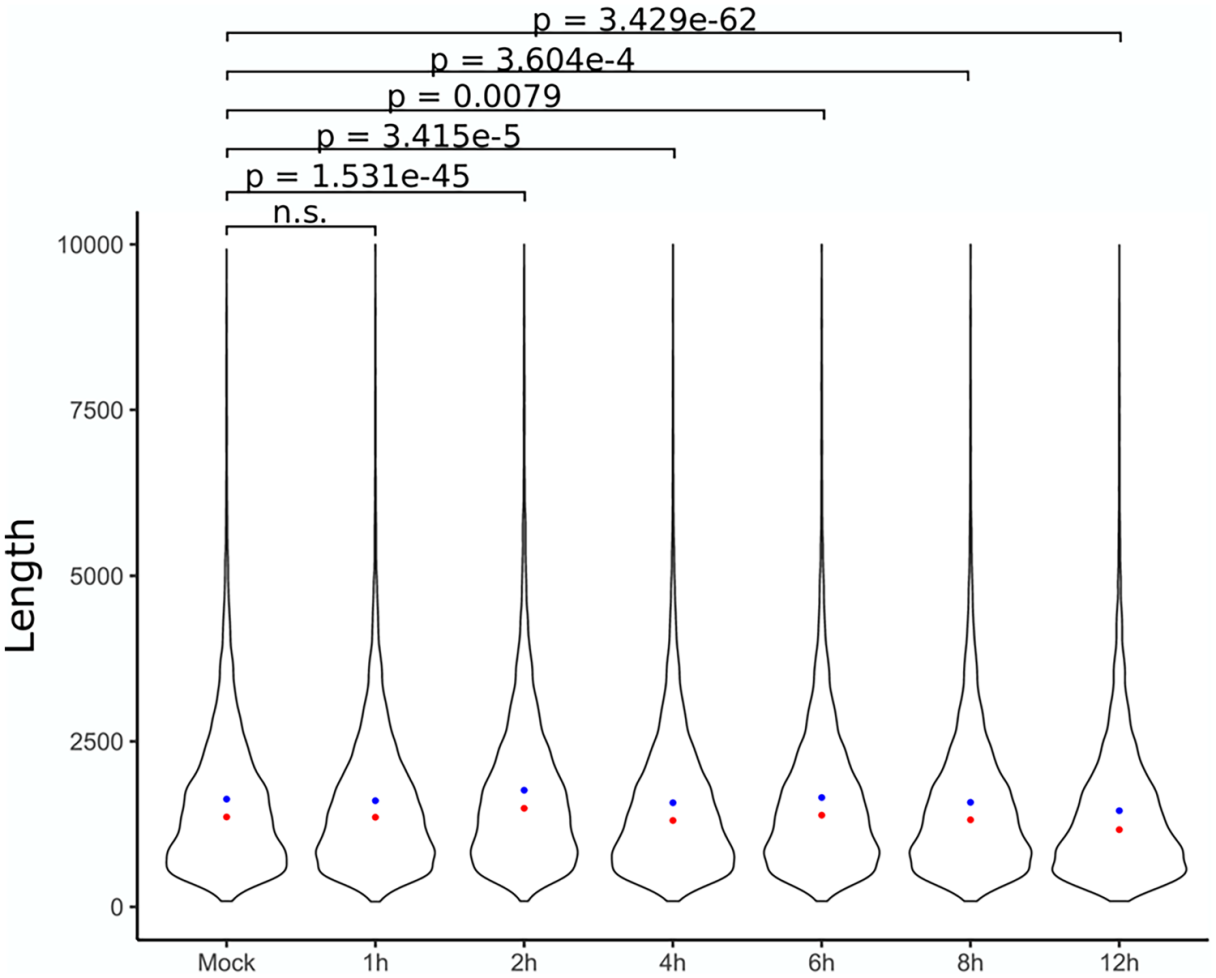
The length of polyadenylated bovine transcripts. Blue dots represent the median while red dots represent the mean of datasets. The figure was created using R v. 3.6.3 (https://www.r-project.org/) and ggplot2 v. 3.3.3 (https://ggplot2.tidyverse.org/reference/ggplot.html) and Inkscape v. 0.92 (https://inkscape.org/).

A previous work reported the disruption of transcript termination in the host caused by HSV-1 infection, resulting in extensive transcriptional overlaps between adjacent gene products^22^. According to our results, the length of polyadenylated transcripts has significantly changed starting from the second hour of the infection. The most pronounced increase in transcript length can be seen at 2 h p.i. (${\bar{\text{x}}}$ change of 134bps, *p* < 0.05), while the greatest decrease was detected at 12 h p.i. (${\bar{\text{x}}}$ change of − 193 bps, *p* < 0.05) compared to the mock samples (${\bar{\text{x}}}$ = 1365 bps). At the interim time points the median transcript length exhibited a fluctuating pattern (at 4 h a ${\bar{\text{x}}}$ change of 53 bps, at 6 h a ${\bar{\text{x}}}$ change of − 27 bps, while at 8 h a ${\bar{\text{x}}}$ change of 44 bps) (Fig. 3). In order to investigate whether disruption of transcript termination also occurs in BoHV-1-infected bovine cells and results non-polyadenylated transcripts, we carried out ONT sequencing based on random oligonucleotide-primed RT, and the obtained dataset was used for the analysis of transcription activity at the intergenic regions. Despite this library yielding a comparable measure of reads mapping to *Bos taurus* (n = 2,222,987), we were unable to detect any substantial amount of fragments mapping to the intergenic regions. Using LRS, we were able to differentiate between TSS isoforms. We detected 80 transcripts with upstream and 142 with downstream TSSs.

### Overall host cell gene expression during the 12 h of virus infection

**Figure 4:**
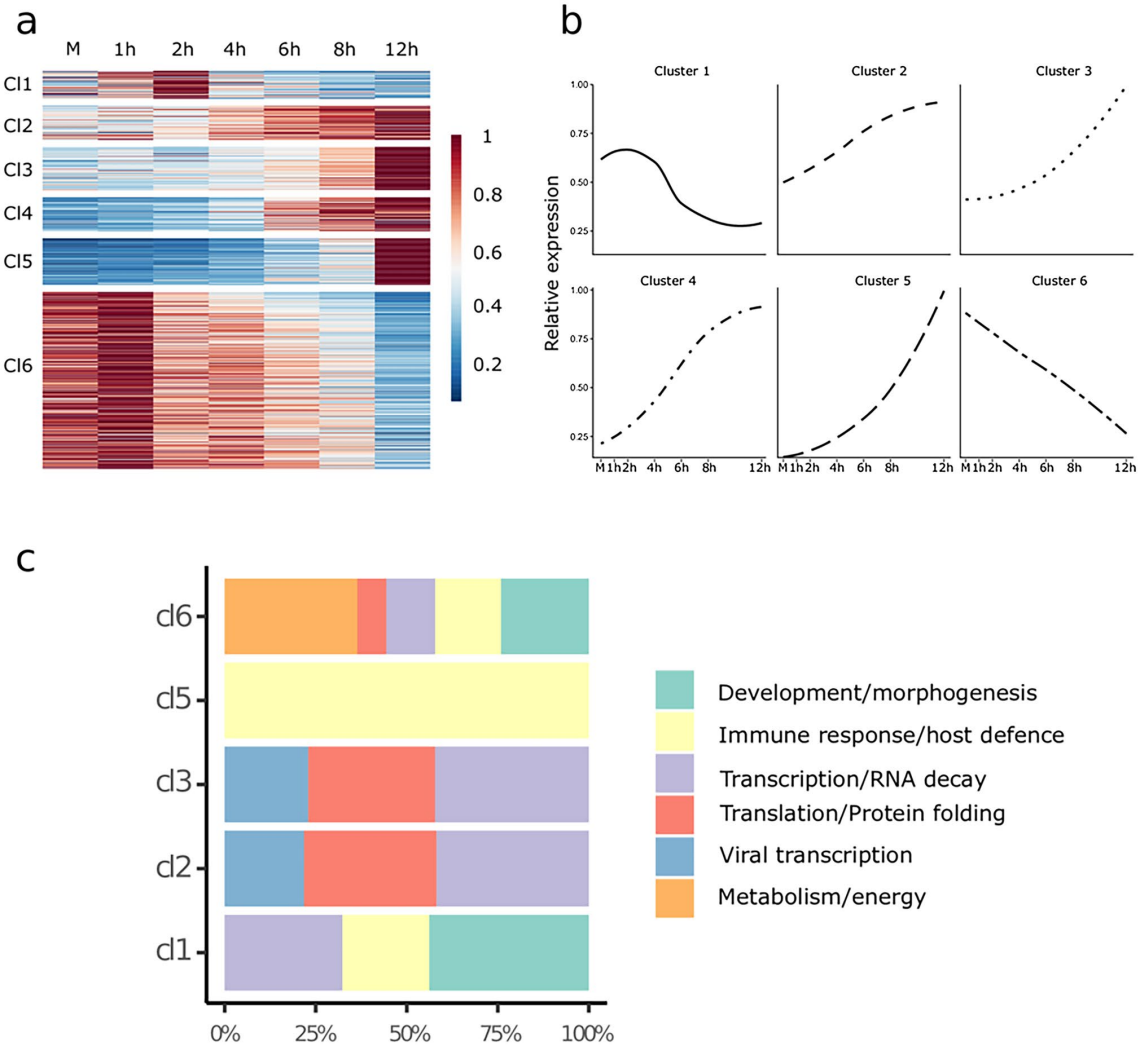
Differential expression analysis of host genes. (**a**) Relative gene expression represented on a heat map. Six distinct kinetic clusters (Cl) were identified among host genes. The scale represents the mean relative gene expression. (**b**) The average change in normalized relative expression of genes present in the six kinetic clusters during infection. (**c**) The distribution of genes associated with the six functional categories according to gene ontology (GO) between the kinetic clusters. The figure was created using R v. 3.6.3 (https://www.r-project.org/); ggplot2 v. 3.3.3^23^ (https://ggplot2.tidyverse.org/reference/ggplot.html); ComplexHeatmap v. 2.2.2 (https://jokergoo.github.io/ComplexHeatmap-reference/book/) and Inkscape v. 0.92 (https://inkscape.org).

This study investigated the effect of viral infection on the cultured bovine cells by a time-course transcriptome analysis using ONT LRS analysis. We carried out direct cDNA sequencing using three biological replicates in each of the six time-points (1 h, 2 h, 4 h, 6 h, 8 h, 12 h) and in the mock-infected sample. We identified a total of 8342 host genes that produced more than ten transcripts in each of the three biological replicates**.** Applying differential expression (DE) analysis with a 0.01 false discovery rate (FDR) threshold, we identified 686 genes among the 8342 host genes that exhibited significantly altered expression levels during the course of virus infection. Genes were clustered by their expression profile and not by their absolute expression levels. In this part of the analysis, we transformed the time series of expression levels to a relative scale representing the expression changes between sampling points. This allowed to cluster the genes by their expression profiles during the course of viral transfection instead of their absolute abundance. We identified six clusters of genes with distinctive expression profiles (Fig. 4a, b and Supplementary Table S2). By analyzing mean expression profiles of gene clusters, we identified four groups of genes (clusters 2–5) that were constantly upregulated, a single group of genes where expression levels were steadily downregulated throughout the entire period of virus infection (cluster 6), and finally, one group that showed initial upregulation followed by downregulation (cluster 1).

We performed an over-representation analysis using the 8342 genes as reference with the PANTHER software tool. We summarized the results of this analysis using GO (Gene Ontology) biological processes and GO molecular functions annotation datasets in Supplementary Table S2 (an FDR < 0.05 was used). Over-represented genes were categorized into six functional groups according to the GO database (Fig. 4c) as follows: 296 genes play a role in cellular metabolism, 257 are involved in transcription and RNA decay, 242 in developmental and morphogenetic processes, 187 in immune response and host defense, 161 in translation and protein folding, whereas 61 genes are specifically associated with in viral transcription related processes.

Genes of the first cluster (n = 53) had medium expression preceding the infection (which was transiently slightly upregulated at the 1 h, and 2 h p.i. time points) followed by downregulation at later measurements. Genes in this cluster were over-represented in pathways controlling a wide variety of developmental and morphogenetic processes. Several genes coding transcription regulatory proteins present in this cluster show diminishing expression throughout the infection. Genes involved in the cytokine regulation of the immune response and inflammatory processes are also affected. The second and third cluster of genes (n = 64, n = 82 respectively) had medium expression preceding the infection that rose at each consecutive time points. The genes of these clusters were over-represented in functions and molecular processes that can be associated with viral gene expression and the virion assembly. An upregulation of genes involved in transcriptional and translational processes, as well as RNA decay was also observed. RNA decay can be an immediate response of the host cell to counteract the accumulation of viral transcripts, or it may be an effort of the virus to eliminate competing host mRNAs in order to facilitate the translation of viral transcripts^24,25^. Some of the over represented genes in these cluster are the members of GO molecular functions that have overlapping sets of genes. For example, the 12 genes (RPS26, RPL5, RPL30, RPS29, RPL31, RPS6, RPL36, RPL37, RPL8, RPS10, RPS21, RPL19) that were significantly upregulated during infection are the members of both the “viral transcription” and the “SRP-dependent co-translational protein targeting to membrane” pathways. Many of these genes are also members of the “nuclear-transcribed mRNA catabolic process, nonsense-mediated decay” pathway.

Genes in the fourth cluster (n = 64) had low relative expression preceding the infection. These genes were upregulated following a sigmoid curve during the infection. Genes in the fourth cluster were not significantly over-represented in any of the GO molecular functions or GO biological processes. The fifth cluster of genes (n = 88) had zero or negligible expression preceding infection but showed an exponential increase in expression during the course of infection. The over-represented genes of this cluster were associated with anti-viral cellular and defense responses such as the type I interferon signaling pathways. The sixth cluster of genes (n = 335) consisted of a huge variety of host genes with high expression preceding viral infection that showed sharp downregulation during the infection. These genes were over-represented in pathways associated with protein folding, cell cycle regulation and mitochondrial processes including aerobic respiration.

### Key response host genes

We performed DE analysis (with FDR = 0.01) on the Mock and 1-h expression values to describe the immediate response of host cells. We identified 6 bovine genes that were significantly down regulated and 19 genes that were significantly up regulated in the three biological replicates (Table 1). Over expression analysis revealed no significant association to either of the GO biological processes or GO molecular functions using the subset of up and down regulated genes or the whole set of genes. However, STRING association analysis revealed 4 networks between these genes. The first gene network (GADD45B, GADD45A, DDIT3, ATF3, IFRD1, CARM1, SQSTM1) contains genes that are associated to host DNA damage response, transcription regulation. Furthermore, one gene plays a role in selective automacrophagy. The second network consists of two of the interferon gamma stimulated genes; IRF9, a transcription factor that plays an essential role in anti-viral activity and MT2A, a metallothionein protein. The third network consists of two genes (SRSF5 and HNRNPDL) associated with pre-mRNA processing, transport and splicing regulation. The cytokine IL11which regulates hematopoietic cells was part of the fourth network. We found IL11 to be down-regulated. In contrast, CXCL5, a gene associated with neutrophil activation and also present in network 4, was up-regulated following virus infection. The remaining four (LASP1, HDAC7, SLC44A2 HSPG2) out of 6 down- regulated genes and eight (ID2, HMGN3, TMEM190, TSC22D1, PRKAR2B, LOC100847759, LOC100847143, LOC100174924) out of 19 up-regulated genes include signaling, transcriptional regulator, developmental genes.

**Table 1:** Up- and downregulated bovine genes in response of viral infection.

Gene	logFC	logCPM	F	*p* value	FDR	A Mock	A 1 h	A 2 h	A 4 h	A 6 h	A 8 h	A 12 h	B Mock
LASP1	− 1.603	7.534	25.183	3.22E−06	2.68E−03	23.42	14.10	30.48	35.71	28.13	27.23	69.40	50.27
HDAC7	− 1.543	6.646	22.935	2.74E−06	2.54E−03	24.54	6.75	13.89	12.13	12.54	6.98	29.46	23.32
CARM1	− 1.502	6.245	15.415	1.11E−04	4.65E−02	11.73	5.62	17.47	13.48	10.97	9.30	5.55	18.85
SLC44A2	− 1.292	6.672	16.843	5.35E−05	2.63E−02	28.01	9.64	14.85	15.38	21.75	18.07	8.26	28.72
HSPG2	− 1.181	8.739	17.042	1.26E−04	4.85E−02	73.59	46.38	68.00	76.82	82.47	58.73	31.34	127.45
IL11	− 1.145	7.292	16.439	6.87E−05	3.19E−02	31.56	15.26	72.50	21.98	4.79	4.76	26.81	44.88
PRKAR2B	5.413	4.788	15.010	1.34E−04	4.85E−02	0.00	4.89	2.33	1.89	6.15	2.52	0.33	0.00
DDIT3	2.729	6.686	41.826	1.08E−09	8.94E−06	3.54	21.28	11.61	5.37	6.40	22.23	17.68	5.39
IRF9	2.648	5.658	14.900	1.41E−04	4.85E−02	1.18	5.97	9.10	3.56	4.65	26.30	9.19	1.80
TSC22D1	2.582	7.034	38.345	2.14E−09	8.94E−06	2.39	20.10	32.15	7.04	20.21	11.41	16.77	6.30
GADD45A	2.057	6.715	28.559	1.91E−07	3.18E−04	3.56	23.38	8.06	7.02	14.02	18.19	22.52	7.18
CXCL5	1.727	7.452	29.238	1.39E−07	2.90E−04	14.00	34.66	42.34	18.98	12.54	7.05	5.52	6.26
LOC100174924	1.650	7.675	24.507	2.44E−06	2.54E−03	16.32	32.22	23.16	18.94	28.14	62.75	107.37	10.76
SRSF5	1.630	7.249	24.298	1.43E−06	1.70E−03	11.67	31.88	40.02	18.95	18.68	15.69	40.50	11.67
IFRD1	1.611	7.468	16.111	8.08E−05	3.55E−02	8.15	14.02	13.39	18.81	31.23	78.46	172.07	1.76
MT2A	1.538	7.832	25.980	9.59E−07	1.33E−03	11.73	43.15	38.09	47.80	25.03	20.72	9.98	26.96
GADD45B	1.534	7.406	22.150	4.04E−06	3.06E−03	9.38	24.93	21.11	12.17	35.79	33.81	39.22	9.86
LOC100847143	1.505	8.114	17.505	1.37E−04	4.85E−02	24.49	42.70	23.01	22.45	39.01	71.76	65.48	19.74
LOC100847759	1.427	9.291	30.238	9.06E−08	2.52E−04	49.00	87.55	72.80	80.38	82.58	141.86	216.50	37.69
ATF3	1.421	6.627	14.844	1.45E−04	4.85E−02	11.62	27.55	13.47	3.61	7.96	20.24	34.46	8.07
TMEM190	1.352	7.169	17.749	3.44E−05	1.92E−02	15.16	26.56	12.80	13.89	20.28	22.34	7.87	11.66
HMGN3	1.300	7.304	17.452	3.96E−05	2.06E−02	12.86	30.23	26.94	18.97	15.69	22.59	8.98	19.77
SQSTM1	1.266	7.963	20.451	9.10E−06	5.42E−03	21.03	46.80	59.72	46.16	25.00	27.07	9.70	17.94
ID2	1.265	8.106	21.046	6.81E−06	4.37E−03	27.98	52.08	41.91	22.30	51.43	45.32	57.00	25.13
HNRNPDL	1.148	9.144	21.068	6.74E−06	4.37E−03	53.68	104.19	86.67	83.79	84.15	103.59	94.65	55.65

Gene	B 1 h	B 2 h	B 4 h	B 6 h	B 8 h	B 12 h	C Mock	C 1 h	C 2 h	C 4 h	C 6 h	C 8 h	C 12 h
LASP1	5.40	8.80	17.40	39.01	30.62	52.60	57.81	22.77	28.37	21.45	36.46	29.21	47.09
HDAC7	8.89	17.13	15.90	14.64	9.51	20.66	35.24	10.70	16.28	22.92	14.01	7.30	2.12
CARM1	5.64	2.83	5.87	14.62	7.90	13.22	19.75	5.28	17.87	5.91	10.03	17.36	18.87
SLC44A2	8.27	19.38	5.93	17.09	15.39	5.22	19.87	12.35	17.71	17.77	13.38	14.60	2.00
HSPG2	45.97	19.97	84.90	60.99	70.68	62.40	172.12	63.24	90.40	54.97	65.03	50.20	33.23
IL11	11.17	31.19	5.93	7.32	7.56	26.49	46.58	27.25	42.11	12.00	7.30	5.47	38.12
PRKAR2B	4.04	2.96	6.99	6.06	5.53	0.20	0.00	2.32	2.84	2.01	0.96	2.73	3.18
DDIT3	46.93	9.66	10.15	9.79	13.49	18.18	4.28	15.81	7.55	17.84	16.40	8.20	10.63
IRF9	8.14	8.90	5.74	1.28	4.22	10.36	0.06	6.93	10.55	4.71	4.53	9.12	7.08
TSC22D1	28.51	60.43	11.56	14.66	6.06	3.26	2.92	21.68	39.73	15.61	14.53	16.45	10.39
GADD45A	24.35	15.73	11.53	12.21	17.32	16.27	7.05	23.76	8.51	12.53	11.17	20.08	39.67
CXCL5	31.57	48.42	28.83	11.02	13.17	17.90	18.26	50.14	52.29	37.98	18.02	7.29	12.20
LOC100174924	60.14	27.63	21.67	31.73	30.82	50.16	12.76	24.04	10.33	32.82	44.95	31.03	40.26
SRSF5	37.17	35.74	31.62	12.24	6.07	5.89	8.56	26.16	32.53	24.50	17.39	14.61	3.84
IFRD1	22.30	21.67	8.80	37.80	40.34	68.65	9.78	16.35	13.31	22.93	39.27	31.93	66.35
MT2A	49.29	17.76	40.35	30.50	34.50	26.18	11.42	52.93	46.65	38.68	41.58	49.31	31.11
GADD45B	48.75	31.34	15.94	25.64	26.89	31.58	16.86	21.59	21.97	31.19	29.70	27.38	27.94
LOC100847143	91.92	44.82	50.39	39.05	40.32	75.89	18.43	33.05	21.91	63.83	54.47	31.02	50.62
LOC100847759	163.93	70.04	95.09	81.71	114.80	175.93	45.20	82.76	56.50	158.07	116.74	94.02	165.44
ATF3	16.38	16.42	5.82	23.09	13.56	16.17	4.38	19.63	9.44	8.73	10.57	15.51	22.65
TMEM190	39.10	34.36	20.24	17.10	11.70	2.89	12.76	29.48	25.40	37.21	22.46	13.69	1.95
HMGN3	31.92	28.30	24.55	17.11	15.51	3.19	7.22	36.11	28.26	38.44	27.02	22.82	10.21
SQSTM1	48.73	43.13	37.53	31.70	13.71	13.29	26.79	52.51	84.56	57.73	29.15	30.13	18.94
ID2	61.03	41.71	15.98	63.38	63.04	70.70	12.86	40.92	39.27	35.01	46.50	52.03	55.76
HNRNPDL	136.17	140.92	138.16	89.02	86.11	44.96	42.44	81.53	85.76	123.50	120.66	71.19	45.41

We detected significant upregulation in 19 and 6 downregulation in 6 host genes in all of the three biological replicates.

In this part of the study, we validated the differential expression of 10 host genes as a result of virus infection using real-time RT-PCR. The expression of the given genes was measured in two different cell populations: mock-infected and infected cells which produced larger up- or downregulation for the particular gene. The differences in transcripts levels between the infected and mock-infected samples are shown in Supplementary Fig. S2. The qPCR data validate our findings that we reached using an LRS approach in each examined gene. We found the expression of the genes ARC, DDIT3, FAM102A, GADD45G, MYLIP and TSC22D1 to be increased in virus-infected cells, while expression levels of the genes ANXA1, C15H11orf74, LOC112446408 and SLC44A2 were decreased compared to mock-infected cells. R values shown in Table 2 indicate the means of the three replicates.

**Table 2:** Real-time RT-PCR analysis of 10 *Bos taurus* transcripts.

Gene	R	Gene	R
ANXA1 (6)	0.045	GADD45G (5)	19.441
ARC (5)	33.221	LOC112446408 (6)	0.654
C15H11orf74 (6)	0.055	MYLIP (4)	2.975
DDIT3 (1)	3.729	SLC44A2 (6)	0.016
FAM102A (3)	3.312	TSC22D1 (1)	2.531

The table shows the relative expression ratio (R) values. Names of the downregulated genes are underlined. (The number of the cluster to which the gene belongs is shown in parentheses).

The results obtained in the analysis of host gene expression were also validated using an in silico approach. We selected 30 housekeeping genes exhibiting highly stable expression in different tissues^26^. Although the abundance of these genes varies, it is expected that, in the normalized data, their expression levels will have very high and significant correlation between biological replicates and the samples at different time-points. We calculated the correlation coefficient (R) and the significance between each biological replicate and measurement point (Supplementary Table S3; Supplementary Fig. S3). The fact that the transcript ratios of housekeeping genes are correlated with a high significance represents an internal validation of the reliability of our sequencing technique and data normalization protocol.

## Discussion

High-throughput long-read sequencing approaches are able to read full-length transcripts, and therefore allow a more comprehensive annotation of RNA molecules. LRS-based studies led to the discovery that the transcriptomes are much more complex than previously thought.

In this study, we annotated a large number of bovine transcripts and analyzed the effect of viral infection upon host gene expression. We found no significant change in the usage of promoters or PASs of the host genes. However, we observed an altered usage of transcript length and splice isoforms of the host RNA molecules. This indicates a modulation of cellular mRNA turnover. The analysis of TSS isoforms suggests that viral infection may have an effect on host mRNA translation, potentially through uORFs^27^, or through other cis-acting elements, such as miRNA binding sites of 5’-UTRs. However, downstream TSSs can also result in truncated in-frame ORFs, which might code for N-terminally truncated polypeptides^28,29^. Unlike in HSV-1-infected cells^22^, we found no increase in the extent of transcriptional readthroughs in BoHV-1-infected-cells.

Based on the alteration of expression kinetics, we detected six distinct gene clusters that had significantly changed expression during the course of virus infection. Based on the overrepresentation analysis of these clusters we distinguished three functional groups. Genes involved in basic cell functions, including morphogenesis, cell cycle regulation, signaling, catabolic pathways and aerobe respiration, are generally downregulated during viral infection. On the other hand, we observed a considerable upregulation of genes involved in antiviral response. Additionally, genes playing a role in transcription, RNA decay, translation and protein folding were also upregulated. Our analysis shows that most of these genes are associated to distinct molecular functions and biological processes indicating general response to virus infection. However, the rest of the unassociated genes could also be associated with either susceptibility to or defense against viral infection. We also identified a small set of immediate response genes that exhibited significantly altered expression 1 h after viral infection.

Altogether, our data provides valuable resources for future functional studies and for understanding how the virus can overcome host defense mechanisms. Furthermore, these results may be helpful for the development of novel antiviral therapies.

## Materials and methods

### Cells and viruses

Madin Darby Bovine Kidney (MDBK, purchased from American Type Culture Collection) cells were infected with the Cooper isolate of Bovine Herpesvirus 1.1. (GenBank Accession JX898220.1). Cells were incubated at 37 °C in a humidified incubator with 5% CO_2_, and were cultured with Dulbecco’s modified Eagle’s medium (DMEM) supplemented with 5% (v/v) fetal bovine serum, 100 µg/mL streptomycin and 100 U/mL penicillin. Cells were either mock-infected or infected with Cooper isolate of BoHV-1 at a multiplicity of infection (MOI) of 5 plaque-forming units (PFU)/cell, incubated at 4 °C for one hour for synchronization of infection, and then placed in an incubator at 37 °C and 5% CO_2_. Infected cells were collected at 1, 2, 4, 6, 8, and 12 h post infection (HPI). Each time-point and mock infection consisted of three replicates (n = 3). Cells were washed with phosphate buffered saline (PBS), scraped from the culture plate and centrifuged at 300 RPM for 5 min at 4 °C.

### RNA isolation

RNA from infected and uninfected cells (MDBK cells) was extracted using the NucleoSpin RNA kit (Machery-Nagel, Bethlehem, PA, USA), with the lysis step augmented by the addition of proteinase K (final concentration 0.37 mg/mL).

### Poly(A) RNA selection and rRNA depletion

For the analysis of the polyadenylated RNAs, the RNA fraction was enriched using Oligotex mRNA Mini Kit (Qiagen). To obtain potential non-polyadenylated transcripts, rRNA depletion was performed using Ribo-Zero Magnetic Kit H/M/R (Epicentre/Illumina).

### ONT non-amplified cDNA sequencing

Direct cDNA libraries were prepared from the mock and six BoHV-1 p.i samples in three replicates using the ONT’s Direct cDNA Sequencing Kit (SQK-DCS109) according to the manufacturer’s instructions. The first cDNA strand synthesis was performed using Maxima H Minus Reverse Transcriptase (Thermo Fisher Scientific) with SSP and VN primers (supplied in the kit) and 100 ng of poly(A) + RNA for each sample. This was followed by the removal of potential RNA contamination using RNase Cocktail Enzyme Mix (Thermo Fisher Scientific), and second strand synthesis using LongAmp Taq Master Mix (New England Biolabs). Double stranded cDNA ends were repaired using NEBNext End repair /dA-tailing Module (New England Biolabs). This was followed by ligation of sequencing adapter employing the NEB Blunt /TA Ligase Master Mix (New England Biolabs). Libraries were barcoded using Native Barcoding (12) Kit (ONT) according to the manufacturer’s instructions.

### RT with oligo(dT) primers

50 ng of poly(A) + RNA was reverse transcribed using SuperScript IV Reverse Transcriptase and oligo(dT) primers (supplied in the kit). The cDNA samples were subjected to PCR using KAPA HiFi DNA Polymerase (Kapa Biosystems) and Ligation Sequencing Kit Primer Mix. End repair and sequencing adapter ligation were carried out as described for the dcDNA-Seq library preparation.

### RT with random oligonucleotide primers

Fifty ng of ribodepleted RNA was reverse transcribed using SuperScript IV Reverse Transcriptase. Additionally, we used a custom-made primer mix composed of a random hexamer sequence and another which is complementary to the Ligation Sequencing Kit Primer (supplied in the kit). PCR, end repair and sequencing adapter ligation were identical to the oligo(dT)-primed RT library. The obtained libraries were barcoded using the 1D PCR Barcoding (96) Kit from Oxford Nanopore Technologies, according to the manufacturer’s instructions. The random oligonucleotide-based cDNA sequencing was primarily used for the identification TESs of the host transcripts.

### LoopSeq single-molecule synthetic long-read sequencing

LoopSeq libraries were prepared from multiplexed 2 h and 12 h post-infection samples in three replicates using the LoopSeqTM Transcriptome 3 × 8-plex Kit. Phasing mRNA protocol was performed as recommended by the manufacturer.

### Purification of libraries

Libraries were purified after each enzymatic step using Agencourt AMPure XP magnetic beads or in the case of dRNA-Seq, RNAClean XP beads (both from Beckman Coulter). Qubit RNA BR and HS Assay Kits and Qubit DNA HS Assay Kit (Thermo Fisher Scientific) were used to measure the total RNA, poly(A) + RNA, and cDNA concentrations, respectively.

### Sequencers

Sequencing of the ONT dcDNA libraries was performed on R9.4.1 SpotON Flow Cells (ONT). To avoid barcode cross-talk from later time points, mock-infected, 1 h and 2 h p.i. samples were sequenced separately from other samples. The LoopSeq library was sequenced on a v2 300 flow cell on the Illumina MiSeq system.

### Pre-processing and data analysis

The MinION data was basecalled using the Guppy basecaller v. 3.4.1. with --qscore_filtering. Reads with a Q-score larger than 7 were mapped to the *Bos taurus* GCF_002263795.1_ARS-UCD1.2 reference genomes using the “-ax splice -Y -C5” options in the minimap2 software^30^ (detailed mapping statistics: Supplementary Table S4). LoopSeq short read data were assembled into long reads using the manufacturer’s web service (https://analysis.loopgenomics.com/accounts/login/), and mapped to the host genome using Minimap2. TSSs, TESs and introns were annotated using our LoRTIA software suite (https://github.com/zsolt-balazs/LoRTIA). Transcripts represented by less than 10 reads were excluded from further analysis. We compared the genomic position of TSSs, TESs and splice junctions of transcripts annotated from the infected samples, to those of the mock control samples. A TSS or a TES was considered “downstream” or “upstream” of the control TSS or TES, if it was located more than 10 bp from the control position, whereas “unaffected”, if it was within 10 bp from the control. If a transcript had splice junctions located at different position than the splice junction in the control, the transcript was considered “alternatively spliced”. If the given transcript was not present in the mock infected samples, but was found in the infected samples the transcript was named “not in the control”.

The sequences of the TSSs and TESs and their neighboring sequences were extracted with our in-house script (https://github.com/moldovannorbert/seqtools), using the previously annotated positions and the reference genome as source. To assess the change in transcript lengths, we first log_10_-transformed the data and tested for variation using the Kruskal–Wallis test. To detect those time points where transcript lengths significantly differed from the those in the mock sample, pairwise two-sided Mann–Whitney U tests were performed between the mock and the infected samples followed by multiple test correction using Bonferroni’s method.

### Analysis of host cell gene expression

In order to assess the effect of the infection on host gene expression, we used the cfDNA-seq results. We excluded MAPQ = 0, secondary and supplementary alignments from all downstream analysis. The reads aligned to the host genome were associated to host genes according to the GCF_002263795.1_ARS-UCD1.2_genomic.gff genome coordinates. Only reads matching the exon structure of the host reference genes (using a + /− 5 base pair window for matching exon start and end positions) were counted. The three biological replicates were grouped in EdgeR^31^ (DGEList function) by the corresponding time points. We normalized the date (calcNormFactors function) with the method = ”TMM” options and we used the robust = True option in the downstream analysis (estimateDisp, glmQLFit, and glmQLFTest functions). Since we had mock, 1 h, 2 h, 4 h, 6 h, 8 h, 12 h measurements, in our model, we tested for DE against mock expression for each time point using data from three biological replicates. To detect genes with significantly changed expression levels (decideTests function), we applied a 0.01 false discovery rate (FDR) threshold, with *p*-values adjusted by the Benjamini & Hochberg procedure (p.adjust function with method = ”BH” option).

Medians of normalized pseudo-counts of DE genes were exported from edgeR^31^ (Table 1). Gene expression levels were normalized to maximal expression levels and were then compared to each other by cluster analysis to reveal which genes had similar expression kinetics during viral infection. Genes were clustered using the amap_0.8-16 R package Kmeans function with the Euclidean distance method. Based on the Calinski criteria, our dataset had an optimal cluster number of 6. The six clusters of genes were visualized via ggplot2 with the geom_smooth function using the median of the relative gene expressions of genes for each time points in each of the identified gene clusters. Using the identified subset of genes, we also performed overrepresentation analysis for each cluster using the number of expressed genes as reference via the PANTHER (version 14.1 using the 2018_04 dataset release)^32^ software tool. We summarized the results of our over-representation analysis (FDR < 0.05) using the Gene Ontology (GO) biological processes and GO molecular functions annotation datasets.

Schematic representation of the workflow is shown in Supplementary Fig. S1.

### Primer design

To validate the effect of viral infection on host gene expression, reverse transcription-based quantitative real-time PCR (qRT-PCR) was used. The gene specific primers of the 10 *Bos taurus* genes which had altered expression levels due to BoHV-1 infection were designed using the PrimerQuest™ Tool (Integrated DNA Technologies, IDT) (Supplementary Table S5). The latest GenBank assembly (GCA_002263795.2) was used as a reference sequence. Primers were purchased from IDT.

### Reverse transcription

RNA samples were used to produce single stranded cDNAs with gene specific primers. Reverse transcription (RT) reactions were performed using 6 ng of RNA and gene-specific primers (0.1 µM final concentration) with SuperScript IV RT enzyme (Invitrogen). The RT reactions were carried out according to the SuperScript IV manual: briefly, the RNA, primer and dNTP containing mixtures were denatured at 65 °C for 5 min, cooled down to 4 °C, then the buffer, the DTT and the RT enzyme were added. RNaseOUT (Invitrogen) was used to avoid RNA degradation. The samples were incubated at 50 °C for 10 min. The reactions were stopped by raising the temperature to 80 °C for 10 min. First-strand cDNAs were diluted tenfold with UltraPure™ DNase/RNase-Free Distilled Water (Invitrogen), then subjected to real-time PCR analysis.

### Quantitative PCR

qPCR experiments were performed using a Rotor-Gene Q cycler (Qiagen). Reactions were carried out in 20 μl reaction mixtures containing 7 μl of cDNAs, 10 μl of ABsolute QPCR SYBR Green Mix (Thermo Scientific) and 3 μl primer mixture (1 μl of forward and 1 μl of reverse primers, 50 nM final concentration, each), as was previously published^33^. The running conditions were as follows: (1) 15 min at 95 °C, followed by 35 cycles of 94 °C for 25 s (denaturation), 60 °C for 25 s (annealing), and 72 °C for 6 s (extension). For those experiments where primer dimer formation was detected, we applied an extra extension step in every PCR cycle with an elevated temperature (between the Tm of the specific product and the Tm of the primer dimers) for 2 s for detection (details are summarized in Supplementary Table S6). The 28S rRNA was used as loading control (reference gene).

### Calculation of relative expression ratios

Relative expression values were calculated according to the following equation, as published in our previous articles^33^:

$$ R = \frac{{E^{{Ct}} gene_{i} /E^{{Ct}} gene_{m} }}{{E^{{Ct}} ref_{i} /E^{{Ct}} ref_{m} }}, $$

where R is the relative expression ratio; E is the efficiency of amplification; Ct is the cycle threshold value; gene refers to any particular gene at the most down- or upregulated time points in virus-infected cells (i) compared to mock-infected (m) samples; and ref is the 28S housekeeping gene, which was used as a reference gene. Average Ct values with their standard deviance (SD) values, amplification efficiencies with SDs and the examined time points are shown in Supplementary Table S6. The relative copy numbers of mRNAs were calculated by normalizing cDNAs to 28S rRNA gene using the Comparative Quantitation module of the Rotor-Gene Q software (Version 2.3.5, Qiagen), which automatically calculates the qPCR amplification efficiency and the take-off points sample-by-sample. Thresholds were set automatically by the Rotor-Gene software. For each gene, 3 replicates were carried out for statistical confidence, and the median of these values along with the standard deviances was calculated.

## Supplementary Information

Supplementary Information 1.

Supplementary Table S1.

Supplementary Table S2.

Supplementary Table S3.

Supplementary Table S4.

Supplementary Table S5.

Supplementary Table S6.

Supplementary Data Item.

## Data Availability

The sequencing datasets generated during this study are available at the European Nucleotide Archive’s SRA database under the accession PRJEB33511 (https://www.ebi.ac.uk/ena/browser/view/PRJEB33511).

## Code availability

The LoRTIA software suite is available on GitHub: https://github.com/zsolt-balazs/LoRTIA. Other scripts used: https://github.com/moldovannorbert/seqtools.
