# Supplementary material for: Time course profiling of host cell response to herpesvirus infection using nanopore and synthetic long-read transcriptome sequencing
Source: Sci Rep. 2021 Jul 9;11:14219. doi: 10.1038/s41598-021-93142-7 (PMC8270970; doi:10.1038/s41598-021-93142-7)
Supplement: Supplementary file 1 — Supplementary Information 1. [file 41598_2021_93142_MOESM1_ESM.docx]

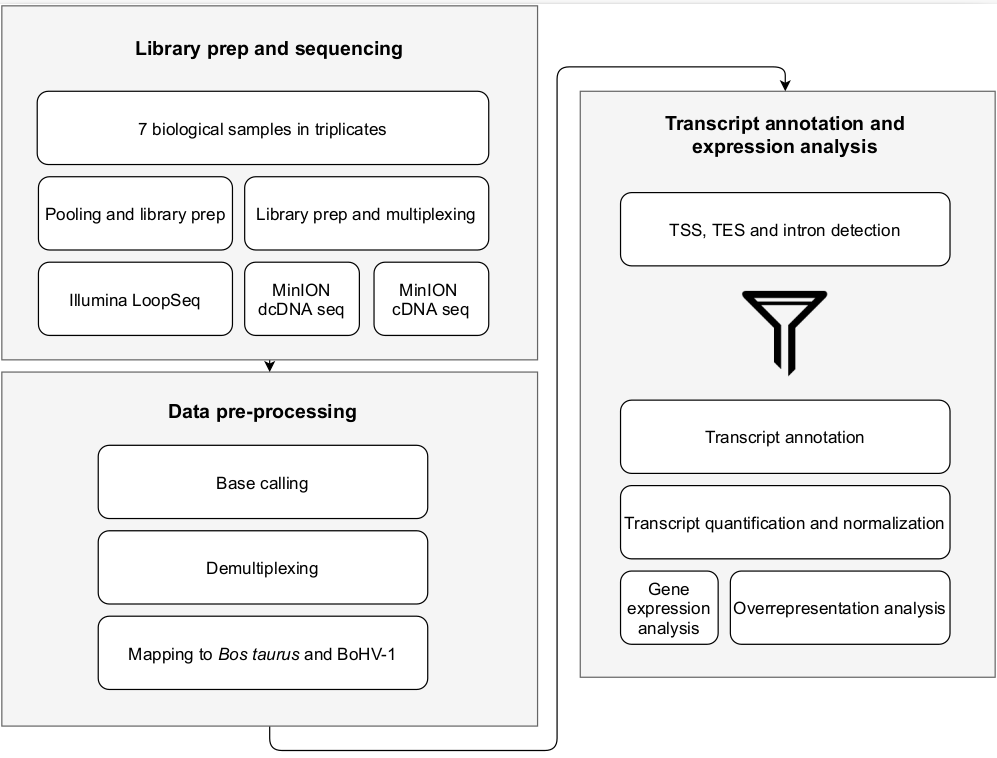


**Supplementary Figure S1. The workflow used to sequence and analyze the data.** The figure was created using the online tool diagrams.net v. 14.7.3 (<https://www.diagrams.net/>).


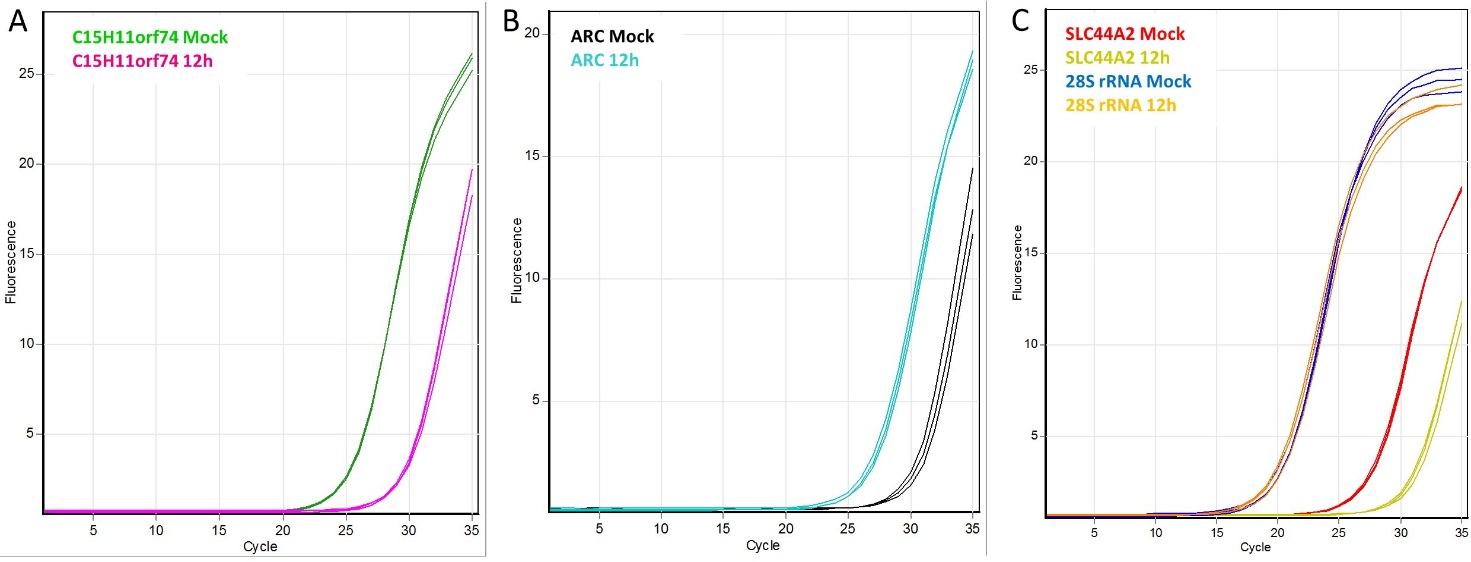


**Supplementary Figure S2. qPCR amplification plots of selected *Bos taurus* genes.** (A) Amplification curves of gene C15H11orf74. BoHV-1 infection has a drastic inhibitory effect on its expression. (B) The expression level of the ARC gene shows significant increase after viral infection. (C) This figure shows the 28S rRNA housekeeping gene (there is no effect of viral infection on its transcriptional activity) and the SLC44A2 gene (the virus exhibits a down-regulatory effect on the expression of this gene). Amplification curves were generated by the software of the real-time PCR cycler (Qiagen, Rotor-Gene Q software version 2.3.5, <https://www.qiagen.com/ie/resources/resourcedetail?id=9d8bda8e-1fd7-4519-a1ff-b60bba526b57&lang=en>)


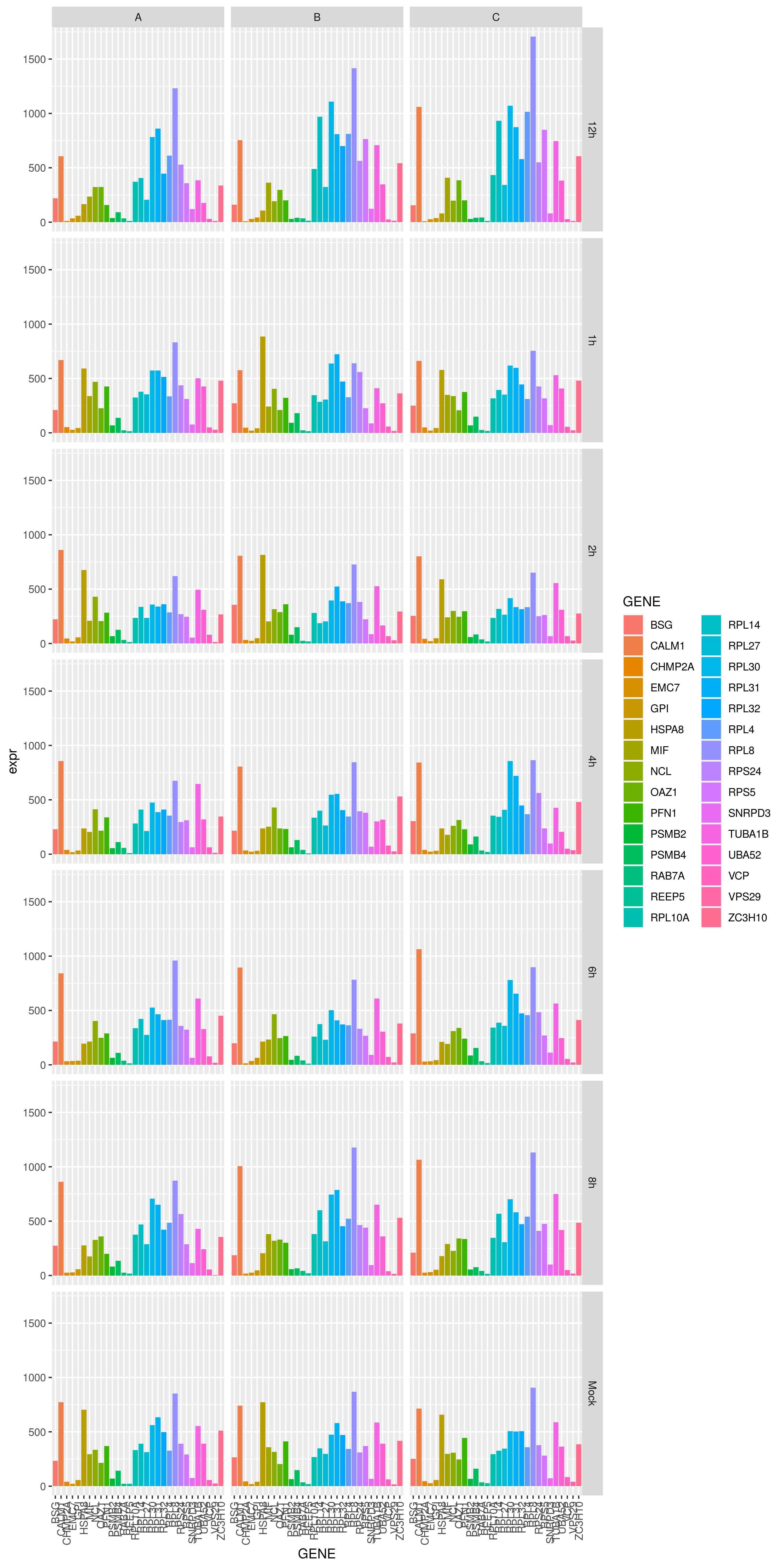


**Supplementary Figure S3. Normalized gene expression of 30 housekeeping genes of biological replicates and different measurement points (biological replicates in columns A, B, C).** The illustration was made by using ggplot2 [23] (<https://ggplot2.tidyverse.org>).

**Supplementary Data Item 1. Transcript annotations of the MDBK cell line.** The positions in the annotation files correspond to the GeneBank assembly with the accession number GCF_002263795.1.

**Supplementary Table S1. TSSs, TESs and introns detected by the LoRTIA software suite.**

**Supplementary Table S2. Genes of the host cell line with distinct expression.**

**Supplementary Table S3. Correlation of the expressions of 30 House-keeping genes between biological replicates and time points**

**Supplementary Table S4. Mapping statistics.**

**Supplementary Table S5. Primers used for qRT-PCR.** Primers labeled with bold letters were used for reverse transcription.

**Supplementary Table S6.** Average of take-off (Ct) values and standard deviances based on three separated reactions. The table also shows the information about the applied extra extension temperatures.
